# Digital Orthodontic Assessment of Mandibular Morphology Using Orthopantomograms: Correlation and Symmetry Analysis of Bilateral Gonial Angles, Bigonial Width, and Bilateral Ramus Heights

**DOI:** 10.3390/jcm14228099

**Published:** 2025-11-15

**Authors:** Adelina Popa, Andra-Alexandra Stăncioiu, Alexandru Cătălin Motofelea, Horia Câlniceanu, Amalia Catalina, Atena Galuscan, Roxana Oancea, Magda Mihaela Luca, Andrada-Nicoleta Nikolajevic-Stoican, Silviu Brad, Camelia Szuhanek

**Affiliations:** 1 Orthodontic Research Center ORTHO-CENTER, Discipline of Orthodontics I, Faculty of Dental Medicine, “Victor Babes” University of Medicine and Pharmacy Timisoara, 9 No., Revolutiei Bv., 300041 Timisoara, Romania; popa.adelina@umft.ro (A.P.); amalia.casalean@umft.ro (A.C.); cameliaszuhanek@umft.ro (C.S.); 2 Center for Molecular Research in Nephrology and Vascular Disease, Discipline of Nephrology, Department VII/Internal Medicine II, Faculty of Medicine, “Victor Babes” University of Medicine and Pharmacy, 300041 Timisoara, Romania; alexandru.motofelea@umft.ro; 3 Department of Periodontology, Faculty of Dental Medicine, Anton Sculean Research Center for Periodontal and Peri-Implant Diseases, ‘’Victor Babes’’ University of Medicine and Pharmacy, 300041 Timisoara, Romania; calniceanu.horia@umft.ro; 4 Clinic of Preventive, Community Dentistry and Oral Health, “Victor Babes” University of Medicine and Pharmacy, Eftimie Murgu Sq. No. 2, 300041 Timisoara, Romania; galuscan.atena@umft.ro (A.G.); roancea@umft.ro (R.O.); 5 Translational and Experimental Clinical Research Centre in Oral Health, Department of Preventive, Community Dentistry and Oral Health, University of Medicine and Pharmacy “Victor Babes”, 300040 Timisoara, Romania; 6 Department of Pediatric Dentistry, Faculty of Dental Medicine, “Victor Babes” University of Medicine and Pharmacy Timisoara, Eftimie Murgu Square 2, 300041 Timisoara, Romania; luca.magda@umft.ro; 7 Faculty of Dental Medicine, “Victor Babes” University of Medicine and Pharmacy, Eftimie Murgu Sq. No. 2, 300041 Timisoara, Romania; nicoleta.stoican@umft.ro; 8 2nd Department of Radiology, Faculty of Dental Medicine, “Victor Babes” University of Medicine and Pharmacy, 300041 Timisoara, Romania; brad.silviu@umft.ro

**Keywords:** artificial intelligence, bigonial width, bilateral gonial angles, dentistry, digital analysis, gonial angle, mandibular asymmetry, mandibular measurements, orthodontics, orthopantomogram, panoramic radiography, ramus height

## Abstract

**Background/Objectives**: Precise evaluation of mandibular morphology is essential for orthodontic diagnosis, growth assessment, and treatment planning. While lateral cephalograms are traditionally used for angular measurements, orthopantomograms (OPGs) allow side-specific assessment of mandibular structures. This study aimed to analyze bilateral gonial angles, bigonial width, and ramus heights on OPGs using artificial intelligence (AI)–assisted tracing, with a focus on symmetry, sex-related differences, and clinical applicability. **Methods:** A cross-sectional study was conducted on 78 Romanian patients (46 females, 32 males; mean age 22.8 ± 8.7 years) seeking orthodontic treatment. Standardized OPGs were obtained and analyzed using WebCeph AI-driven software. Bilateral gonial angles, bigonial width, and ramus heights were measured. Symmetry between sides and differences between sexes were evaluated, and correlation analyses were performed. Inter-rater reliability was assessed using intraclass correlation coefficients (ICCs). **Results:** The mean right and left gonial angles were 126.3° and 127.1°, respectively, with no significant sex- or side-related differences (*p* > 0.05). Bigonial width averaged 134.9 mm, being slightly larger in males, although not statistically significant (*p* = 0.240). Ramus heights were comparable bilaterally (right: 48.4 mm; left: 48.3 mm), with males showing slightly greater values. Correlation analysis demonstrated strong bilateral symmetry for gonial angles (r = 0.795) and ramus heights (r = 0.895). Negative correlations were observed between gonial angles and both bigonial width and ramus height, whereas bigonial width correlated strongly with ramus height. Measurement reproducibility was high (ICC > 0.75). **Conclusions:** OPGs, when combined with AI-assisted analysis, provide reliable and reproducible measurements of gonial angles and ramus heights, comparable to cephalometric values. Their ability to assess right and left sides separately enhances clinical utility in detecting asymmetries and monitoring mandibular growth. However, caution is advised when interpreting transverse parameters such as bigonial width, where CBCT remains the gold standard. AI-driven OPG analysis represents a cost-effective, accessible, and low-radiation diagnostic tool with significant potential for precision orthodontics.

## 1. Introduction

The right diagnosis and treatment plan rely on information gathered from diagnostic tools like X-rays, clinical exams, and study models. In dentistry, panoramic radiography is the usual way to diagnose problems and plan treatments. Both dentists and orthodontists use it. It tells us a lot about the teeth and the bone that supports them. It is used to check for cysts, cancer, extra teeth, teeth that are missing from birth or fall out early, teeth that are fused to the bone or retained in an abnormal way, the path of tooth eruption, bone pathology, and mandibular asymmetry. It is smart to attain a correct diagnosis of asymmetries before starting treatment; in this context, you can talk about the limitations of treatment and your therapeutic choices [1]. Most doctors do not use panoramic images to find out if someone has a mandibular asymmetry, but some experts do [2,3]. While Mattila et al. say that lateral cephalography is a better way to find the gonial angle, panoramic radiography is better because it lets you measure the right and left gonial angles separately, without having to add them together [4].

Extraoral digital dental imaging is slowly becoming the norm in the field of dentistry. Because digital radiography images can be saved and retrieved at any time, they are an important tool in the area of forensic anthropology. There are many types of radiographic pictures that can be used to find out someone’s age and gender. These include digital imaging, lateral cephalograms, panoramic radiographs, lateral oblique X-rays, and more advanced imaging technologies like CBCT (cone-beam computed tomography) and MRI. Orthopantomograms (OPGs) are the extraoral X-rays that dentists most often use to look at the structures of the maxillo-mandibular complex [5].

An orthopantomogram (OPT) is a radiographic picture that is made from a tooth panoramic tomography. Dentists and maxillofacial surgeons often ask for this view to check the teeth, gum bone support, maxillary and mandibular bony structures, temporomandibular joints, and maxillary sinuses. It is also one of the two standard X-rays that our school asks for when looking into mandibular fractures. General radiologists look at these images and write official reports that are given to the doctors in the emergency room [6].

In cases of asymmetry, like hemifacial microsomia and unilateral condylar hyperplasia, it is important to obtain accurate measurements of the structures on both sides in order to identify and plan any kind of surgery or distraction osteogenesis. Lateral cephalograms, on the other hand, cannot be used for this reason reliably. Since Paatero discovered the panoramic method in 1952, dentists have used it in a wide range of dental fields. The doctor can acquire a full picture of the maxillofacial complex with panoramic radiographs while being exposed to less radiation. Panoramic radiography is an important diagnostic tool because it can show the whole stomatognathic system, including the teeth, cheeks, temporomandibular joints, and sinuses. In orthodontics, it is often used to show important details about the teeth, such as their axial inclinations, maturation times, and the tissues around them. With OPG, it is easy to see the structures on the right and left sides separately, without having to look at structures that overlap or are stacked on top of each other, like on a lateral cephalogram. But measures taken on panoramic X-rays have been called into question because of big mistakes in the way they were performed [7].

Because panoramic X-rays are common, they can be used to look at the changes in shape that come with becoming older and to see if there are any differences or similarities between men and women. Several studies have used panoramic X-rays to find out the gonial angle, the height of the ramus, and the width of the bigonial bone [8,9,10,11,12].

OPG is one of the most important diagnostic tools in orthodontics. It is used to plan treatment before it starts and to see how well or poorly the treatment worked by checking for specific teeth, their root length, axial inclinations, morphology, and structure, as well as their eruption sequence and spatial relationships. In 1991, Levandoski was the first person to study panoramic radiographs. Since then, not many studies have been performed on the topic because quantitative measurements are thought not to be true because these radiographs have a magnification that can change infinitely [13].

To acquire exact measurements of the length and angle of mandibles, panoramic X-rays can be repeated [14].

This research examines bilateral mandibular characteristics using the OPG modality to characterize symmetry and covariation patterns. We do not see OPG as a substitute for CBCT in the transverse plane; instead, we evaluate the internal consistency and clinical applicability of OPG-derived values, considering bigonial width as exploratory and advising the use of CBCT for exact transverse assessment.

Comprehending mandibular morphology is crucial for orthodontic diagnosis, growth assessment, and treatment planning. Although cephalometric radiography has traditionally been the primary technique for angular and linear evaluation of the mandible, orthopantomograms (OPGs) are more accessible, cost-effective, and expose patients to less radiation. While several studies have compared cephalometric and panoramic measurements of the mandible, most have focused mainly on the gonial angle, leaving the reliability of OPG-based assessments of ramus height and bigonial width uncertain. Moreover, few studies have used artificial intelligence–assisted landmark identification in panoramic analysis.

Therefore, the current study sought to evaluate mandibular morphology using AI-assisted OPGs, emphasizing bilateral symmetry, correlation, and proportional analysis of the gonial angle, ramus height, and bigonial breadth. The research aimed to assess the reliability of these measurements within the OPG modality and to highlight their potential use as supplementary tools in orthodontic diagnosis.

## 2. Materials and Methods

### 2.1. Ethical Considerations

The research was carried out on patients from the Discipline of Orthodontics I, Faculty of Dental Medicine, “Victor Babeș” University of Medicine and Pharmacy, Timișoara. All participants provided written informed consent prior to inclusion, and the study protocol received ethical approval from the Scientific Research Ethics Committee of the “Victor Babeș” University of Medicine and Pharmacy, Timișoara, Romania (CECS Nr. 04/26 January 2024).

### 2.2. Study Design and Participants

This analytical cross-sectional study was designed to evaluate specific mandibular parameters measured on orthopantomograms (OPGs), with the primary objectives of identifying potential correlations among these variables and comparing measurements between the left and right sides of the mandible. By examining bilateral gonial angles, bigonial width, and ramus heights, the analysis aimed to determine whether significant asymmetries or proportional differences existed, thereby contributing to a better understanding of mandibular morphology in the studied population.

#### Sample Size Determination

The sampling framework and determination of study size are described below.

The study included 78 panoramic radiographs, selected retrospectively based on image quality and completeness of diagnostic records during the study period. Although no formal a priori power analysis was performed due to the retrospective nature of the design, the sample size was comparable to or greater than that used in similar studies [1,15,16,17], ensuring adequate statistical power for the intended comparisons.

The effect size was reported in a previous study [15].

All participants were Romanian patients who presented for orthodontic treatment at the Clinic of Orthodontics I, Faculty of Dental Medicine, “Victor Babeș” University of Medicine and Pharmacy, Timișoara.

Eligibility for inclusion was determined based on the following criteria: age appropriate for reliable mandibular growth assessment; maintenance of good oral and periodontal health; Romanian nationality; and written informed consent.

Exclusion criteria included: prior orthodontic or orthognathic treatment, congenital craniofacial anomalies, systemic diseases requiring hormonal therapy, endocrine or neurological disorders, maxillofacial trauma, incomplete records, or low-quality/distorted radiographs.

### 2.3. Procedure Methodology

#### 2.3.1. Protocol and Measurements for Digital Orthopantomogram

All patients’ orthopantomograms were acquired with their heads positioned in a natural head posture, serving as a standardized reference during image capture [18].

During OPG acquisition, patients were positioned standing upright with shoulders relaxed, back straight, and feet together to ensure postural stability. The head was aligned such that the median sagittal plane was perpendicular to the floor and the Frankfort horizontal plane was parallel to the ground. The chin was gently placed on the chin rest, and the patient was instructed to bite on a disposable bite block to stabilize the mandible and separate the dental arches. The tongue was positioned against the hard palate throughout the exposure to minimize air space artifacts in the maxillary region. Radiographs were immediately evaluated for image clarity, correct patient alignment, absence of movement artifacts, and proper exposure parameters. Any images not meeting diagnostic quality standards were repeated to ensure the accuracy of subsequent digital measurements.

OPGs and, where applicable, panoramic radiographs were acquired using a PaX-i3D Green imaging unit (Vatech, Hwaseong, Republic of Korea) operated by certified radiographic technicians. The device was calibrated according to the manufacturer’s specifications before each imaging session to ensure consistent image quality. Standard exposure parameters were selected based on patient size and age, in accordance with the “as low as reasonably achievable” (ALARA) radiation safety principle. All images were stored in DICOM format for subsequent digital analysis.

WebCeph (version 1.0.3; webceph.com) is an artificial intelligence–driven, web-based platform designed for orthodontic and orthognathic analysis, allowing both automated and semi-automated landmark identification and measurement on digital radiographs. In this study, the software was employed exclusively for the analysis of orthopantomograms (OPGs).

All OPG images were exported from the imaging unit in high-resolution JPEG format, downloaded, and stored on a Lenovo IdeaPad 5 Pro laptop (Lenovo Group Ltd., Beijing, China) before processing. Each radiograph was checked for clarity, optimal contrast, correct patient positioning, and absence of artifacts that could compromise measurement accuracy. The panoramic radiographs had a resolution of 3000 × 2400 pixels (96 dpi, 24-bit depth).

Once uploaded to WebCeph, the AI algorithm automatically detected key anatomical landmarks relevant to the gonial angles, bigonial width, and ramus height measurements. The semi-automatic mode was also utilized, allowing manual verification and adjustment of landmark positions by an experienced orthodontist to ensure precision. All measurements were performed under standardized viewing conditions, using calibrated monitor settings and controlled ambient lighting to minimize glare and visual fatigue.

Several researchers have used WebCeph for landmark detection [19,20,21].

A calibration factor of 65 pixels was applied for all linear measurements performed on the panoramic radiographs [20]. The digital images, saved in JPEG format, were imported into WebCeph, an AI-powered orthodontic analysis platform patented by both the Korean and United States Intellectual Property Offices.

All analyses were performed on a 14-inch display to maintain a consistent viewing scale. Prior to import into WebCeph, each greyscale radiographic file was downloaded from the imaging system and stored on a Lenovo IdeaPad 5 Pro laptop (Lenovo Group Ltd., Beijing, China) under standardized file management and storage conditions.

On the panoramic radiographs, the following measurements were recorded: bilateral gonial angles (right and left), bigonial width, and mandibular ramus heights (right and left).

The anatomical landmarks and measurement definitions used in this study followed standard cephalometric and panoramic radiographic conventions [4,7,15,17]. Gonial angle, bigonial width, and ramus height were defined as detailed below, and the same definitions were applied consistently in all figures and digital tracings obtained from WebCeph.

Gonial angle (left and right)—measured as the angle formed between a line along the lower border of the mandible passing through the condyle and a line along the posterior border of the ascending ramus. The mean gonial angle obtained from panoramic radiograph measurements was 122.74° (SD = 1.00°) overall, 122.84° (SD = 0.80°) for males, and 122.74° (SD = 1.00°) for females [1].

Bigonial width—The bigonial width represents the linear distance between the right and left gonion points, measured horizontally across the mandible. The gonion (Go) is defined as the most inferior, posterior, and lateral point at the external angle of the mandible. In this study, bigonial width was determined as the direct distance between the left and right gonion landmarks on panoramic radiographs. The mean bigonial width was 193.31 mm (SD = 13.52 mm) in females and 217.63 mm (SD = 10.87 mm) in males [16].

Ramus height (left and right)—The ramus height was measured as the linear distance from the most superior point on the mandibular condyle to the lowest point on the inferior border of the mandible. The mean right ramus height was 4.75 mm (SD = 0.65 mm) in males and 4.70 mm (SD = 0.48 mm) in females. The mean left ramus height was 4.81 mm (SD = 0.57 mm) in males and 4.73 mm (SD = 0.48 mm) in females [22].

Gonial angle: The gonial angles were measured following the method described by Mattila et al. A line was digitally drawn on each panoramic radiograph tangential to the most inferior point at the gonial region and along the lower border of the mandibular body. A second line was traced tangential to the posterior borders of the ramus and the condyle. The intersection of these two lines defined the gonial angle, which was measured on the right or left side, depending on which provided the clearest image.

Bigonial width: The bigonial width was defined as the horizontal distance between the right and left gonion (Go) points. The gonion was identified as the most inferior, posterior, and lateral point on the external angle of the mandible [14].

All measurements were conducted twice by the same examiner and verified by a second examiner to ensure consistency. The data were recorded in millimeters and degrees, and descriptive and inferential statistical analyses were subsequently performed.

#### 2.3.2. Reliability Analysis

To verify measurement accuracy, both intra- and inter-examiner reliability were evaluated. Measurements were taken by two examiners, and 20% of the dataset was re-measured after two weeks. The intraclass correlation coefficient (ICC) was computed using a two-way random-effects model with absolute agreement; values above 0.75 indicated strong reliability.

#### 2.3.3. Measurement Validity Safeguards

To alleviate panoramic-specific distortion, we executed (i) standardized positioning (natural head posture; FH parallel to the floor) and rigorous quality control with repeated acquisitions when standards were not satisfied; (ii) device-level scaling within WebCeph and a constant pixel factor for linear measurements; (iii) AI-assisted landmark detection in semi-automatic mode with manual expert verification and adjustment; and (iv) inter-rater reliability assessed as two-way random-effects, absolute-agreement, single-measures ICC (2,1) with 95% confidence intervals. These measures seek to guarantee intra-modality repeatability of OPG measurements, recognizing that CBCT serves as the benchmark for transverse accuracy.

#### 2.3.4. Statistical Analysis

Statistical tests were performed using SPSS version 26.0 (IBM Corp., Armonk, NY, USA). The Shapiro–Wilk test was used to assess normality. For normally distributed variables, paired *t*-tests were applied to compare left and right sides; otherwise, the Wilcoxon signed-rank test was used. Correlations between parameters were assessed using Pearson’s or Spearman’s correlation coefficients, depending on data distribution. The level of statistical significance was set at *p* < 0.05.

**Figure 1:**
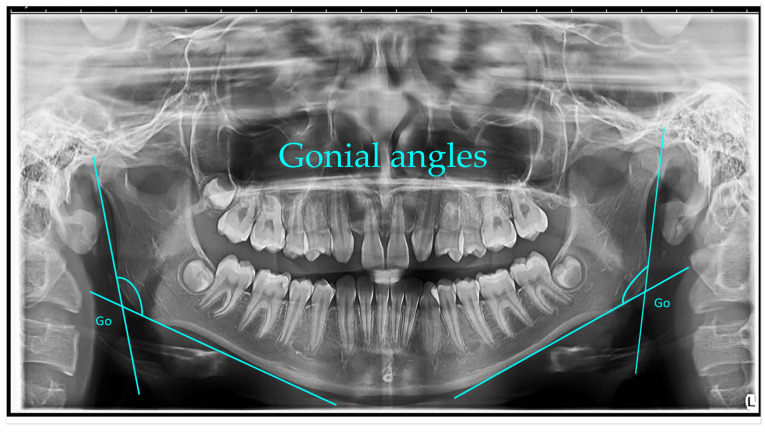
Bilateral Gonial Angle Measurement on OPG. Go (gonion)—the most posterior, inferior, and lateral point on the mandibular angle. Lines were drawn along the posterior border of the ramus and the lower border of the mandible, intersecting at the gonial angle (°). Both right and left gonial angles are displayed for comparative analysis. Note: Images represent direct screenshots exported from the WebCeph^®^ AI platform. Landmarks and measurement lines were automatically generated and manually verified by the authors.

**Figure 2:**
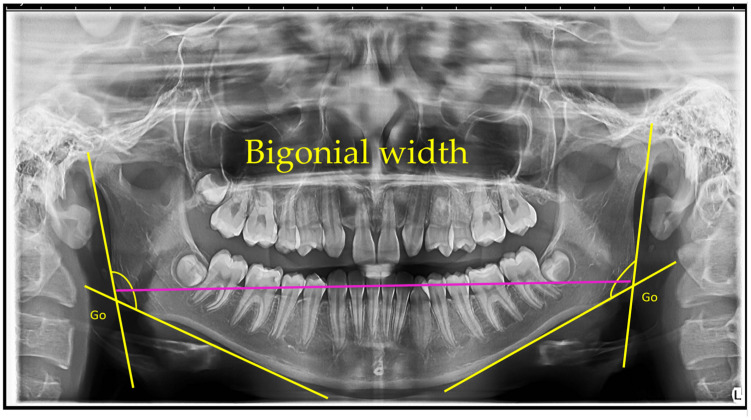
Bigonial Width Measurement on OPG. Go (gonion)—the most posterior, inferior, and lateral point of the mandibular angle. The magenta line connects the bilateral Go points, indicating bigonial width (mm). Note: Images represent direct screenshots exported from the WebCeph AI platform. Landmarks and measurement lines were automatically generated and manually verified by the authors.

**Figure 3:**
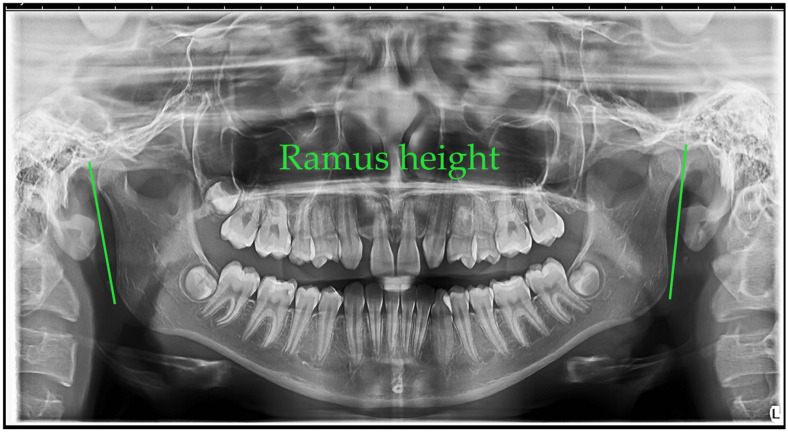
Bilateral Ramus Height Measurement on OPG. Co (condylion)—the most superior point on the mandibular condyle; Inf—the most inferior point on the mandibular border. The green line (Co–Inf) denotes ramus height measured bilaterally for comparative analysis. Note: Images represent direct screenshots exported from the WebCeph^®^ AI platform. Landmarks and measurement lines were automatically generated and manually verified by the authors.

**Figure 4:**
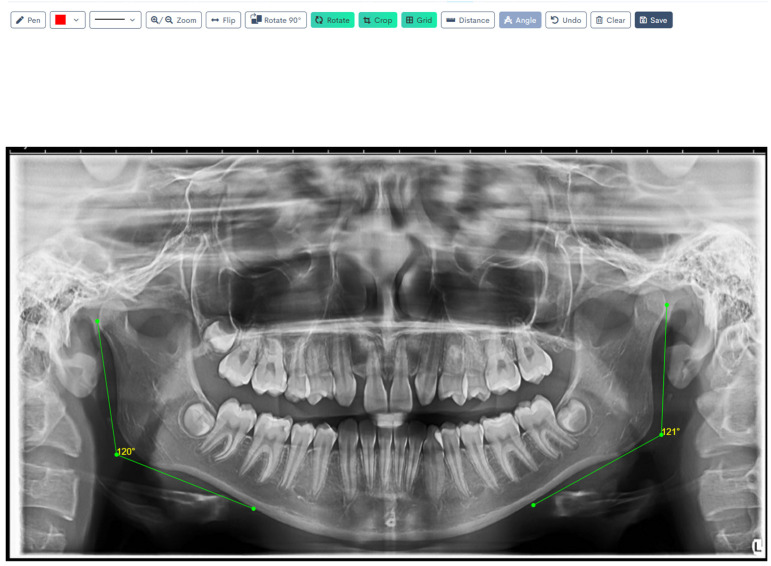
Digital Tracing of Bilateral Gonial Angles in WebCeph. Go (gonion)—the most posterior, inferior, and lateral point at the mandibular angle. Tangents drawn along the posterior border of the ramus and the lower border of the mandibular body intersect to form the gonial angle (°). Note: Images represent direct screenshots exported from the WebCeph AI platform. Landmarks and measurement lines were automatically generated and manually verified by the authors.

**Figure 5:**
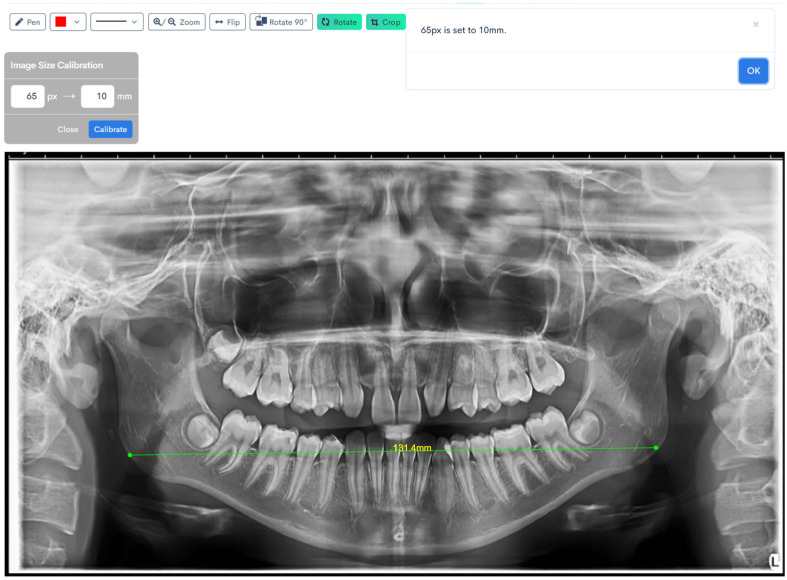
Bigonial Width Digital Tracing on WebCeph. The green line connects the automatically detected bilateral gonion (Go) points, showing the measured bigonial width (mm). Note: Images represent direct screenshots exported from the WebCeph AI platform. Landmarks and measurement lines were automatically generated and manually verified by the authors.

**Figure 6:**
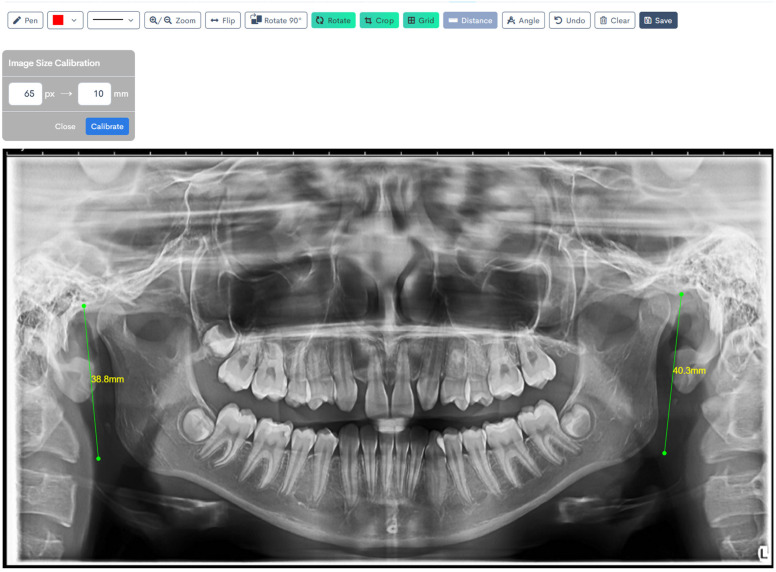
Digital Tracing of Bilateral Ramus Heights in WebCeph. Co (condylion)—the most superior point on the mandibular condyle; Inf—the most inferior point on the mandibular border. Ramus height (Co–Inf) is represented by the green lines with on-screen readouts (left: 38.8 mm; right: 40.3 mm), illustrating bilateral measurement and comparison. Note: Images represent direct screenshots exported from the WebCeph AI platform. Landmarks and measurement lines were automatically generated and manually verified by the authors.

Figure 1, Figure 2, Figure 3, Figure 4, Figure 5 and Figure 6 illustrate the AI-assisted digital measurements exported from the WebCeph software, showing the identification of anatomical landmarks and linear or angular dimensions analyzed in this study.

Panoramic radiograph (orthopantomogram) illustrating the measurement of bilateral gonial angles. The gonion (Go) point was identified on both the right and left mandibular borders, and lines were drawn along the posterior border of the ramus and the lower border of the mandible to form the gonial angle. Both right and left gonial angles are indicated for comparative analysis (Figure 1).

Orthopantomogram showing the measurement of bigonial width. The gonion (Go) points, located at the most inferior, posterior, and lateral aspects of the mandibular angles on both sides, are identified. The bigonial width is determined as the linear horizontal distance between the left and right gonion landmarks, indicated by the magenta line. Yellow lines depict the mandibular borders used to identify the gonion points (Figure 2).

Orthopantomogram illustrating the measurement of right and left mandibular ramus heights. The ramus height was defined as the linear distance from the most superior point of the mandibular condyle to the lowest point on the inferior border of the mandible, shown by the green lines. Measurements were recorded bilaterally for comparative analysis (Figure 3).

Digital tracing of bilateral gonial angles was performed directly in WebCeph on a panoramic radiograph, after which the angles were computed from a line along the posterior border of the mandibular ramus and a line along the inferior border of the mandibular body. The left and right gonial angles (shown in green) are displayed with on-screen angle readouts (e.g., 120° and 121°), representing the final measurements obtained through the program’s digital tracing workflow (Figure 4).

Digital tracing of bigonial width performed on WebCeph using a panoramic radiograph. The measurement represents the horizontal distance between the right and left gonion points (Go), shown by the green line with an on-screen readout of 131.4 mm (Figure 5).

Digital tracing of bilateral ramus heights performed on WebCeph using a panoramic radiograph. Ramus height was defined as the linear distance from the most superior point on the mandibular condyle to the most inferior point on the mandibular border. The left ramus height measured 38.8 mm, and the right ramus height measured 40.3 mm, as shown by the green lines with on-screen readouts (Figure 6).

## 3. Results

The study population comprised 78 participants, with 46 females (59%) and 32 males (41%). The median age was 23 years, with an interquartile range (IQR) of 16 to 27 years, indicating a predominantly young cohort with a relatively narrow age distribution.

Examination of craniofacial parameters demonstrated balanced values across the sample. The median right gonial angle measured 126° (IQR 121–131), while the left gonial angle was 128° (IQR 122–130), suggesting symmetrical angular morphology between sides. Bigonial width showed a median of 134 mm (IQR 128–143), reflecting consistent transverse mandibular proportions within the group.

Vertical mandibular dimensions were comparable bilaterally. The right mandibular ramus height had a median of 48.3 mm (IQR 44.6–51.7), and the left ramus height was similar at 47.9 mm (IQR 43.4–52.6). These findings point to overall harmony in mandibular ramus development, with only minor variations between sides.

In summary, the cohort was predominantly female and relatively young. Craniofacial measurements revealed symmetrical gonial angles and ramus heights, along with consistent bigonial width, underscoring the homogeneity of mandibular morphology within the sample (Table 1).

The study cohort included 78 individuals, comprising 46 females and 32 males. Age distribution differed significantly between groups (*p* = 0.048). Females presented with a mean age of 24.4 years (SD 9.6, range 10.0–55.0), whereas males were younger, with a mean of 20.5 years (SD 6.8, range 7.0–34.0). The overall mean age was 22.8 years (SD 8.7, range 7.0–55.0).

Analysis of gonial angles revealed no statistically significant sex-related differences. The mean right gonial angle measured 125.7° (SD 5.8, range 114.0–140.0) in females and 127.1° (SD 8.2, range 107.0–144.0) in males (*p* = 0.368), yielding a total mean of 126.3° (SD 6.9). Similarly, the mean left gonial angle was 126.9° (SD 6.1, range 116.0–139.0) in females and 127.5° (SD 7.9, range 111.0–145.0) in males (*p* = 0.726), with an overall mean of 127.1° (SD 6.8).

Bigonial width demonstrated a modest but non-significant tendency toward higher values in males. Females exhibited a mean width of 133.6 mm (SD 10.3, range 115.7–157.2), compared with 136.7 mm (SD 12.6, range 99.1–173.1) in males (*p* = 0.240). The overall mean was 134.9 mm (SD 11.4).

**Figure 7:**
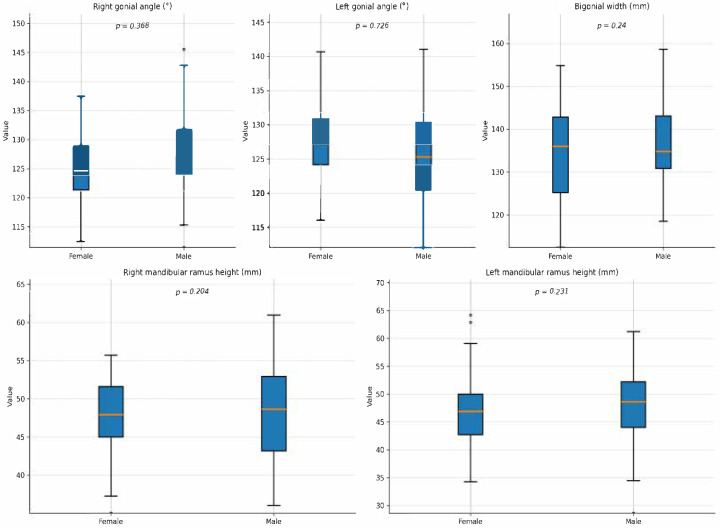
Distribution of craniofacial measurements by gender.

Mandibular ramus height was also greater in males, though differences did not reach statistical significance. On the right side, females had a mean height of 47.7 mm (SD 5.5, range 37.7–57.8), whereas males presented a mean of 49.4 mm (SD 6.3, range 34.0–62.2), with an overall mean of 48.4 mm (SD 5.9, *p* = 0.204). Comparable results were observed on the left side: females demonstrated a mean height of 47.6 mm (SD 5.8, range 38.2–60.0), while males reached 49.3 mm (SD 6.7, range 31.9–62.2), producing a total mean of 48.3 mm (SD 6.2, *p* = 0.231) (Table 2, Figure 7).

Correlation analysis identified several significant associations between age and craniofacial parameters. Age correlated positively with right mandibular ramus height (r = 0.251, 95% CI 0.031–0.449, *p* = 0.026), indicating that increasing age was associated with greater ramus height on the right side. A weaker positive but non-significant association was also observed between age and left ramus height (r = 0.182, 95% CI −0.042 to 0.389, *p* = 0.111). No significant correlations were found between age and gonial angles or bigonial width.

Bilateral gonial angles demonstrated strong symmetry, with a highly significant positive correlation (r = 0.795, 95% CI 0.695–0.864, *p* < 0.001). In contrast, gonial angles correlated negatively with transverse and vertical mandibular measures. The right gonial angle was inversely associated with bigonial width (r = −0.338, 95% CI −0.521 to −0.125, *p* = 0.002) and right ramus height (r = −0.281, 95% CI −0.474 to −0.062, *p* = 0.013). Similarly, the left gonial angle correlated negatively with bigonial width (r = −0.323, 95% CI −0.509 to −0.108, *p* = 0.004) and left ramus height (r = −0.225, 95% CI −0.427 to −0.003, *p* = 0.047). These findings suggest that greater gonial angle size is associated with reduced mandibular width and height.

Bigonial width correlated strongly with right ramus height (r = 0.664, 95% CI 0.518–0.773, *p* < 0.001) and left ramus height (r = 0.641, 95% CI 0.489–0.756, *p* < 0.001), indicating that broader mandibular width was consistently associated with greater vertical development. Bilateral ramus heights were highly correlated (r = 0.895, 95% CI 0.840–0.932, *p* < 0.001), confirming marked mandibular symmetry (Table 3).

### Sensitivity Analysis

The bilateral symmetry and the correlation structure shown in Table 3 remained substantially unchanged.

## 4. Discussion

The present study investigated mandibular morphology using orthopantomograms (OPGs) with AI-assisted landmark detection, focusing on bilateral gonial angles, bigonial width, and ramus heights. Our findings showed no significant side-to-side differences in gonial angles or ramus heights, suggesting overall mandibular symmetry, while bigonial width demonstrated greater variability, particularly in males. These results are broadly consistent with previously published studies, though some differences in values and reliability across modalities have been noted.

A correct assessment of the shape of the mandible is still very important in orthodontics, maxillofacial surgery, and forensic science. Orthopantomograms (OPGs) have become more popular because they can take pictures of both the right and left sides of the jaw separately, are easy to access, and do not require a lot of radiation. Lateral cephalograms were once thought to be the “gold standard” for angular analysis. With the release of systems that use artificial intelligence (AI), like WebCeph, OPGs’ ability to provide repeatable measurements has grown. This work adds to the growing body of evidence by showing that OPGs can accurately measure the gonial angle and ramus height, but not much for horizontal measurements like bigonial width.

In our sample, the mean right gonial angle was 126.3° and the left 127.1°, with no significant sex or side differences. This aligns closely with previous investigations. Radhakrishnan et al. (2017) found nearly identical mean gonial angle values of 122.79° on OPG and 122.74° on lateral cephalograms, confirming strong agreement between the two methods [1]. Similarly, Zangouei-Booshehri et al. (2012) reported OPG means of 127.07° ± 6.10° and cephalometric means of 127.5° ± 6.67°, also demonstrating close correlation [17].

Katti et al. (2016) observed a mean panoramic gonial angle of 121.13° ± 4.95°, with no significant side-to-side or gender differences, supporting our finding of bilateral symmetry [23]. More recently, Uslu-Akçam (2024) compared panoramic and cephalometric values, reporting means of 124.8° (right) and 125.1° (left) on OPGs and 126.4° on lateral cephalograms, with significant correlations across modalities [24]. Rehman et al. also confirmed this consistency, with an OPG mean of 125.05° ± 7.24° in a mixed cohort (male mean: 124.10°, female mean: 125.59°), and noted variations based on vertical facial divergence [25]. Together, these findings indicate that OPGs provide a valid alternative to lateral cephalograms for gonial angle assessment, especially when bilateral evaluation is required.

Several previous investigations have also reported that OPG-derived measurements of gonial angle and ramus height are comparable to those obtained from lateral cephalograms, confirming the reliability and reproducibility of these panoramic measurements [1,17,23,24]. These findings from the literature are consistent with the present results and further support the use of OPGs as a valid modality for assessing mandibular morphology, particularly in bilateral symmetry analysis.

Our study revealed that bigonial width was larger in males (136.7 mm) compared with females (133.6 mm), though differences were not statistically significant. This pattern is in agreement with prior reports. Arthanari et al. (2024) identified highly significant sexual dimorphism, with mean bigonial widths of 217.63 mm in males and 193.31 mm in females (*p* < 0.001) [16]. Similarly, Leversha et al. demonstrated wider bigonial breadth in males, with values decreasing gradually with age [15]. Forensic studies have also reinforced the role of bigonial width in sexual dimorphism. Karmarkar et al. (2023), in a large Indian cohort (n = 600), reported mean widths of 182.5 mm in males and 176.79 mm in females, confirming significant gender-related differences [26]. Although our study showed the same trend, the lack of statistical significance may be attributed to a smaller sample size and population-specific variability. It is also well-established that OPGs are prone to magnification and distortion errors, particularly in the transverse plane, which may reduce the reliability of bigonial width compared with CBCT or direct anthropometry.

We observed slightly greater ramus height in males than in females, though differences were not statistically significant. These results are in line with the findings of Leversha et al., who reported consistently larger ramus heights in males across age groups [15]. This consistency supports the notion that ramus height is both a robust diagnostic measure for orthodontics and a key parameter for forensic age/sex estimation. However, as with bigonial width, subtle variations may be obscured by the inherent distortion of panoramic radiographs.

Beyond descriptive comparisons, correlation analysis revealed several clinically relevant associations. Age correlated positively with right ramus height (r = 0.251; *p* = 0.026), although the left side did not reach significance. This contrasts with the findings of Leversha et al., who reported that increasing age was associated with an increase in gonial angle but a reduction in both ramus height and bigonial width in Australian adults aged 19–69 years [15]. Bhuyan et al. reported age-related increases in gonial angle, ramus height, and bigonial width in an Indian population, underlining the importance of population-specific growth patterns [27].

Rationale for Landmark Transfer—The same cephalometric landmarks—Condylion (Co), Gonion (Go), and inferior mandibular border points (Inf)—were deliberately applied on orthopantomograms (OPGs) to maintain anatomical consistency with traditional lateral cephalometric analysis and to facilitate cross-modality comparison. Although panoramic imaging differs from cephalometry in projection geometry, these reference points can be consistently identified bilaterally on OPGs, allowing reliable correlation and symmetry analysis.

Several previous studies have reported good agreement between OPG-derived and cephalometric values for gonial angle and ramus height, supporting their methodological transfer [4,7,15,17]. However, panoramic imaging is prone to non-linear magnification and geometric distortion, particularly in the transverse dimension, which can affect linear parameters such as bigonial width. Therefore, OPG-based measurements should be interpreted as in-modality estimates rather than absolute anatomical dimensions. Despite these inherent limitations, the use of cephalometric landmarks on OPGs provides clinically meaningful data for mandibular symmetry assessment, offering a practical balance between diagnostic accuracy, accessibility, and radiation safety.

Overall, our findings support the integration of OPG-based digital analysis into orthodontic diagnostics. Gonial angle and ramus height measurements on OPGs are reproducible and comparable to cephalometric values, offering clinicians a cost-effective, accessible, and lower-radiation tool. The ability to measure right and left sides separately provides a clear advantage in diagnosing mandibular asymmetries, such as hemifacial microsomia or unilateral condylar hyperplasia, where side-specific assessment is crucial. At the same time, our study reinforces prior caution that horizontal parameters such as bigonial width should be interpreted carefully on OPGs, with CBCT reserved for cases requiring precise transverse evaluation. AI-assisted platforms such as WebCeph further improve measurement accuracy and reproducibility, helping reduce examiner variability and enhancing clinical confidence.

Our findings support the in-modality (OPG) reproducibility of the gonial angle and ramus height and demonstrate strong bilateral symmetry. Given the intrinsic panoramic magnification and geometric distortion, bigonial width on OPG should be interpreted cautiously and primarily for screening or longitudinal follow-up, not for definitive transverse decisions; CBCT is recommended where surgical planning or precise transverse assessment is required. Accordingly, we have framed bigonial width as an exploratory parameter in the present study.

Clinical Relevance: The use of AI-assisted panoramic analysis in orthodontic diagnosis has several practical benefits. OPG-based analysis allows precise and repeatable bilateral assessments of gonial angle and ramus height, allowing doctors to identify mandibular asymmetry and developmental discrepancies at an early stage, even with standard low-radiation imaging. Although it cannot entirely replace CBCT or cephalometric radiography, it offers a significant supplementary method that improves diagnostic efficacy and minimizes patient exposure to superfluous radiation. In clinical situations when CBCT is contraindicated—such as initial assessment, growth surveillance, or subsequent evaluation—AI-enhanced OPGs may effectively inform clinical decision-making and treatment strategy. Conversely, CBCT is the preferred technique for comprehensive three-dimensional assessment when surgical or structural asymmetry is anticipated. Consequently, AI-assisted OPG analysis enhances rather than replaces sophisticated imaging, according to the ALARA principle, and promotes safer, data-informed orthodontic treatment.

## 5. Limitations and Future Directions

This study presents several issues that require attention. The sample size was relatively small, and there was a higher number of women compared to men. This error may have made it harder to identify differences in men’s and women’s mandibular parameters. It is time for bigger, more diverse studies to prove the patterns that have been seen and come up with strong benchmarks that include everyone in the community. Second, efforts were made to standardize how patients are placed and how radiographs are taken. However, panoramic radiographs can still have errors in magnification and distortion because the patient may move or not be lined up properly. The use of digital calibration and AI to help with tracing makes things more accurate, but these sources of mistakes can still be found and fixed. Using three-dimensional imaging techniques like CBCT to compare OPG-based measures would make their claims stronger. Third, because this study used a cross-sectional design, it is not possible to say exactly how changes in the shape of the jaw happen with age. Longitudinal studies that follow patients through different stages of growth would help find out how the geniomaxillary angle, ramus height, and bigonial width change over time and if OPGs can accurately record these changes. Last but not least, the study only looked at one group of people in Romania. The shape of the jaw varies depending on race and population; hence, researchers should be cautious when applying these results to other groups of people. Future research should compare individuals from different groups to establish standard numbers for orthodontic diagnosis. In the future, it will be very important to use 3D images as the gold standard, obtain bigger samples, and ensure that AI-driven measures are correct across many different groups. This type of work could facilitate the creation of standard panoramic radiography standards. In the long run, such studies would enhance the usefulness of OPGs in both clinical and investigative settings. The goal of future research should be to combine panoramic standards across populations, test AI algorithms on groups of people of different ethnicities, and thoroughly compare OPG-derived measures with CBCT. Such attempts would not only make orthodontic diagnosis more consistent, but they would also make it easier for people all over the world to use OPGs to figure out someone’s age and sex.

The same cephalometric landmarks (Condylion, Gonion, and inferior mandibular boundary points) were deliberately used on OPGs to maintain anatomical consistency with lateral cephalograms and facilitate comparative analysis across modalities. Notwithstanding the projectional and geometric disparities between the two imaging modalities, these landmarks may be consistently recognized bilaterally on OPGs, hence facilitating symmetry analysis. Prior research has shown satisfactory consistency of OPG-derived gonial angle and ramus height measurements in comparison to cephalometric standards [4,7,15,17]. Nonetheless, panoramic imaging presents non-linear magnification and distortion, particularly in the horizontal plane; hence, OPG results should be regarded as in-modality estimations rather than absolute values. This method yields clinically significant data on mandibular proportions and bilateral symmetry, while acknowledging the inherent limits of panoramic imaging.

This cohort did not undergo a pilot study comparing the CBCT and OPG methods; hence, while OPG readings exhibited strong inter-rater reliability, criterion validity against CBCT was not determined in this instance. Future research will conduct a formal agreement analysis (e.g., Bland–Altman and ICC) on a specific CBCT subset to quantify bias and the limits of agreement for bigonial width and ramus height.

## 6. Conclusions

This study assessed mandibular morphology using orthopantomograms (OPGs) with AI-assisted digital tracing, focusing on the correlation, symmetry, and bilateral analysis of gonial angles, bigonial width, and ramus heights.

The findings demonstrated strong bilateral symmetry and significant correlations between mandibular angular and linear parameters. Gonial angles and ramus heights showed consistent and reproducible measurements within the OPG modality, while bigonial width exhibited greater variability, reflecting the known limitations of panoramic imaging for transverse measurements.

Previous studies have reported that OPG-derived measurements of gonial angle and ramus height show close agreement with values obtained from lateral cephalograms, supporting the comparability of these imaging modalities [1,17,23,24]. These results indicate that AI-assisted OPG analysis provides a reliable and accessible method for evaluating mandibular symmetry and proportional relationships, making it a valuable adjunct in orthodontic diagnostics and follow-up. However, transverse parameters such as bigonial width should be interpreted cautiously, and CBCT remains the gold standard for precise three-dimensional assessment, especially in surgical or asymmetry-related cases.

Overall, OPG-based bilateral assessment contributes to a more comprehensive understanding of mandibular morphology while supporting low-radiation, cost-effective diagnostic strategies in orthodontic practice. Future studies should further validate these findings against CBCT and establish normative data across populations. Within OPG, AI-assisted analysis produces dependable bilateral measurements of gonial angle and ramus height for symmetry evaluation and growth tracking. Transverse characteristics, such as bigonial breadth, should be considered exploratory on OPG, with CBCT designated as the gold standard for exact transverse assessment. In clinical practice, OPG serves as the primary, low-radiation imaging modality for routine orthodontic and symmetry evaluation, while CBCT should be reserved only for selected cases that require detailed three-dimensional information—such as craniofacial anomalies, asymmetry, or surgical planning—in full accordance with the ALARA principle.

AI-assisted OPG analysis enhances, rather than substitutes, conventional cephalometric and CBCT assessments. By facilitating bilateral and symmetry-based evaluation with less radiation exposure, it enhances diagnostic confidence and aids in personalized orthodontic planning within a secure and economical diagnostic framework.

This summary highlights the complementary diagnostic value of AI-assisted OPG analysis while maintaining focus on clinical applicability and patient safety.

## Figures and Tables

**Table 1:** Characteristics of the study population.

Characteristic	Total (N = 78)
Gener	
Female	46 (59%)
Male	32 (41%)
Age (years)	23 (16, 27)
Right gonial angle (°)	126 (121, 131)
Left gonial angle (°)	128 (122, 130)
Bigonial width (mm)	134 (128, 143)
Right mandibular ramus height (mm)	48.3 (44.6, 51.7)
Left mandibular ramus height (mm)	47.9 (43.4, 52.6)

**Table 2:** Association of craniofacial parameters by gender.

Variable	Female (N = 46)	Male (N = 32)	Total (N = 78)	*p* Value
Age (years)	24.4 (9.6) [10.0–55.0]	20.5 (6.8) [7.0–34.0]	22.8 (8.7) [7.0–55.0]	0.048
Right gonial angle (°)	125.7 (5.8) [114.0–140.0]	127.1 (8.2) [107.0–144.0]	126.3 (6.9) [107.0–144.0]	0.368
Left gonial angle (°)	126.9 (6.1) [116.0–139.0]	127.5 (7.9) [111.0–145.0]	127.1 (6.8) [111.0–145.0]	0.726
Bigonial width (mm)	133.6 (10.3) [115.7–157.2]	136.7 (12.6) [99.1–173.1]	134.9 (11.4) [99.1–173.1]	0.24
Right mandibular ramus height (mm)	47.7 (5.5) [37.7–57.8]	49.4 (6.3) [34.0–62.2]	48.4 (5.9) [34.0–62.2]	0.204
Left mandibular ramus height (mm)	47.6 (5.8) [38.2–60.0]	49.3 (6.7) [31.9–62.2]	48.3 (6.2) [31.9–62.2]	0.231

**Table 3:** Correlation matrix of age and craniofacial parameters.

Variable	Age	Right Gonial Angle (°)	Left Gonial Angle (°)	Bigonial Width (mm)	Right Mandibular Ramus Height (mm)	Left Mandibular Ramus Height (mm)
Age (years)	—					
Right gonial angle (°)	−0.175 (95% CI −0.382 to 0.050, *p* = 0.126)	—				
Left gonial angle (°)	−0.024 (95% CI −0.245 to 0.199, *p* = 0.833)	0.795 (95% CI 0.695 to 0.864, *p* < 0.001)	—			
Bigonial width (mm)	0.012 (95% CI −0.211 to 0.234, *p* = 0.919)	−0.338 (95% CI −0.521 to −0.125, *p* = 0.002)	−0.323 (95% CI −0.509 to −0.108, *p* = 0.004)	—		
Right mandibular ramus height (mm)	0.251 (95% CI 0.031 to 0.449, *p* = 0.026)	−0.281 (95% CI −0.474 to −0.062, *p* = 0.013)	−0.221 (95% CI −0.423 to 0.001, *p* = 0.051)	0.664 (95% CI 0.518 to 0.773, *p* < 0.001)	—	
Left mandibular ramus height (mm)	0.182 (95% CI −0.042 to 0.389, *p* = 0.111)	−0.223 (95% CI −0.425 to −0.001, *p* = 0.050)	−0.225 (95% CI −0.427 to −0.003, *p* = 0.047)	0.641 (95% CI 0.489 to 0.756, *p* < 0.001)	0.895 (95% CI 0.840 to 0.932, *p* < 0.001)	—

## Data Availability

All data regarding this manuscript can be requested from the corresponding author at andra.stancioiu@umft.ro.
